# Reconstructing secondary test database from PHM08 challenge data set

**DOI:** 10.1016/j.dib.2018.11.085

**Published:** 2018-11-20

**Authors:** Oguz Bektas, Jeffrey A. Jones, Shankar Sankararaman, Indranil Roychoudhury, Kai Goebel

**Affiliations:** a Warwick Manufacturing Group, University of Warwick, Coventry CV4 7AL, United Kingdom; b Data Science and Analytics Manager, Pricewaterhouse Cooper, San Jose, CA 95110, United States; c Stinger Ghaffarian Technologies, Inc., NASA Ames Research Center, Moffett Field, CA 94035, United States; d NASA Ames Research Center, Moffett Field, CA 94035, United States; e Luleå Technical University, Division of Operation and Maintenance Engineering, Luleå, Sweden

**Keywords:** Commercial modular aero-propulsion system simulation, C-MAPPS datasets, PHM08 challenge data set, Data-driven prognostics

## Abstract

In this data article, a reconstructed database, which provides information from PHM08 challenge data set, is presented. The original turbofan engine data were from the Prognostic Center of Excellence (PCoE) of NASA Ames Research Center (Saxena and Goebel, 2008), and were simulated by the Commercial Modular Aero-Propulsion System Simulation (C-MAPSS) (Saxena et al., 2008). The data set is further divided into "training", "test" and "final test" subsets. It is expected from collaborators to train their models using “training” data subset, evaluate the Remaining Useful Life (RUL) prediction performance on “test” subset and finally, apply the models to the “final test” subset for competition. However, the "final test" results can only be submitted once by email to PCoE. Before the results are sent for performance evaluation, in order to pre-validate the dataset with true RUL values, this data article introduces reconstructed secondary datasets derived from the noisy degradation patterns of original trajectories. Reconstructed database refers to data that were collected from the training trajectories. Fundamentally, it is formed of individual partial trajectories in which the RUL is known as a ground truth. Its use provides a robust validation of the model developed for the PHM08 data challenge that would otherwise be ambiguous due to the high-risk of one-time submission. These data and analyses support the research data article “A Neural Network Filtering Approach for Similarity-Based Remaining Useful Life Estimations” (Bektas et al., 2018).

**Specifications table**

**Table:** 

Subject area	*Engineering*
More specific subject area	*Prognostics and Health Management*
Type of data	*Raw, Analyzed*
How data were acquired	*Extracted from PHM08 Challenge Data Set Training Trajectories*[2]
Data format	*Raw data, Reconstructed database*
Experimental factors	*PHM08 Challenge Data Set is divided into ‘training’, ‘test’ and ‘final test’ subsets. The reconstructed secondary test subsets were obtained from ‘training’ subsets by attributing to ‘final test’ subsets.*
Experimental features	*PHM08 Challenge Data Set was carried out using C-MAPSS by simulating turbofan engine degradation*[2]*. The dataset describes how damage propagation can be modeled within the modules of aircraft gas turbine engines simulated under different combinations of operational conditions and fault mode.*
Data source location (of original PHM08 Challenge Data Set)	*The data set was provided by the Prognostics Center of Excellence of NASA Ames Research Center, Moffett Blvd, Mountain View, CA 94035, USA.*
Data accessibility (of original PHM08 Challenge Data Set)	*Data from the challenge competition held at the 1st international conference on Prognostics and Health Management (PHM08) is publicly available at Data Repository of NASA Prognostics Center of Excellence.*
	https://ti.arc.nasa.gov/tech/dash/groups/pcoe/prognostic-data-repository/
Related research article	*O. Bektas, J.A. Jones, S. Sankararaman, I. Roychoudhury, K. Goebel, A neural network filtering approach for similarity-based remaining useful life estimation, Int. J. Adv. Technol. (2018).*

**Value of the data**

• With these data, it is possible to pre-evaluate C-MAPSS prognostics datasets that have been downloaded several thousand times and referred by more than 70 notable papers since its release in 2008 [4].
• The data are useful because they represent a multi-regime response from a high-fidelity simulation of non-linear turbofan engines that closely models real complex systems.
• The variability caused by sensor noise, operational regimes and fault modes accommodate the nature of complex system dynamics.
• Reconstructed data provide multiple training and test trajectories that allow models to learn system behavior.
• Reconstructing of secondary test database allows practical experimental setups for the PHM08 data challenge ranking [2].

## 1 Data

Data from the data challenge competition held at the 1st international conference on Prognostics and Health Management (PHM08) are carried out using C-MAPSS which is a NASA developed tool used to simulate realistic large commercial turbofan engines. It was coded in MATLAB (The MathWorks, Inc.) and Simulink (The MathWorks, Inc.) environments with several editable input parameters that allow users to enter values specific to their own applications regarding operational profile, environmental conditions, etc. [5].

**Fig. 1:**
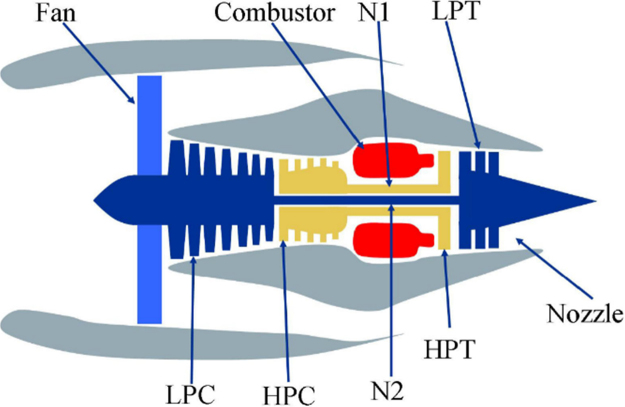
Simplified diagram of a C-MAPSS engine simulation [5].

**Fig. 2:**
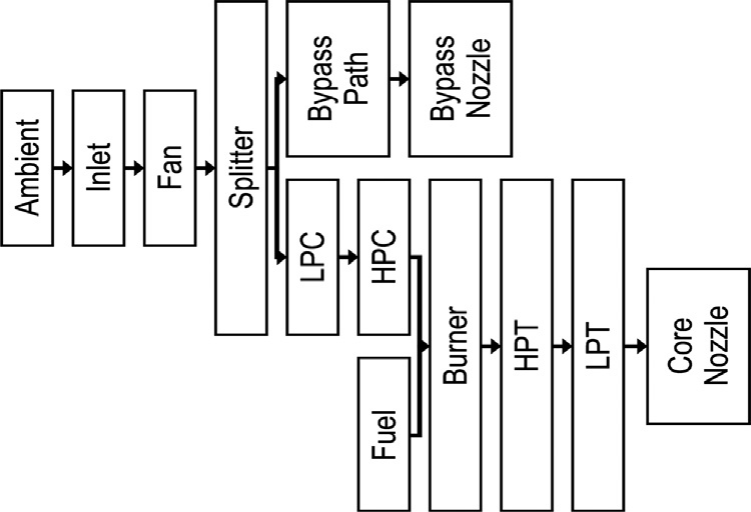
The layout demonstrating the various subroutines of a C-MAPSS engine model [3], [5].

The engine diagram in Fig. 1 shows the basic fragments of a C-MAPSS engine simulation, and the flow chart in Fig. 2 shows how the simulation is formed by the assembly of different subsystems.

In PHM08 Challenge dataset, the generalized time-varying health index equation proposed by Saxena et al. [3] is applied to simulate the damage propagation model

(1) $$h\left(t\right)=1-d-\exp\;(at^{b})$$

where *d* is an arbitrary point in the wear-space, *a* and *b* are model parameters and *t* is time. Since the common degradation models follow the exponential behavior of the fault evolution and similar degradation trends in practice are observed [6], an exponential term while modeling changes of health parameters is used in the dataset.

In general, the performance of a data-driven prognostic method depends on measured condition monitoring data [7], which allows an understanding of system degradation [8]. The features of the PHM08 data challenge include the following characteristics that make it both convenient and suitable for the development of prognostic algorithms on multistep-ahead remaining useful life calculations [2], [4].

• Each subset contains multiple multivariate time series describing sensor magnitudes over time and three different operational settings that indicate variations of regimes.
• The sensors are contaminated with noise that simulate the variability of sensor measurements during operations.
• Each trajectory has a specific initial wear level and manufacturing variation. This wear level is considered normal, and it is unknown to the developers.
• The fault signature is ׳׳hidden׳׳ on account of noise and operational conditions.
• Data sets are divided into training, test and final test trajectories (individual subsets). The training trajectories are implemented to build up the to train remaining useful life prediction algorithms and therefore, the instances are formed of complete run-to-failure data which can be used to feed the test and final test trajectories that are only set up by shorter instances up to a certain time prior to adopted system failure. The main challenge for developers is to estimate remaining useful life of ‘test’ and ‘final test’ subsets by learning from ‘training’ data.
• Each subset is from a different instance of a complex turbofan engine system. The complete dataset can be regarded as representing a fleet of an aircraft of the same type.

The raw readings in the data set are snapshots of the parameters taken during operating conditions of a single cycle, and each column corresponds to a different reading. The first two columns are respectively the trajectory unit number and time of operational cycles, while columns 3, 4 and 5 are operational settings representing the regimes. The remaining are different sensor readings from operations which should be used to identify the health degradation.

PHM08 Challenge Data Set only includes one corresponding training subset for two different testing subsets. The users are expected to model their algorithms using this single set of training data for both ׳test׳ and ׳final test׳ sets provided in the same package. Also, the true RUL values are not provided for the challenge participants. Instead, users are asked to upload their “test” results on the repository page to receive their asymmetrical scoring function results [2]. Since the true RUL numbers are not given, it is not possible to validate the results of individual trajectories with any other prognostic metrics. For the “final test”, users can only send their results once, and therefore this subset does not allow a test-and-trial type of submission.

## 2 Experimental design, materials and methods

Reconstructed databases in this data article were first proposed in [9] and describe the data that were cut out from the original training trajectories. Since the estimated RUL values for PHM08 data challenge can only be submitted once and there are no information to pre-validate the dataset with true RUL [2], the model in [1] is first experiment by reconstructed secondary test databases extracted from the multi-dimensional degradation patterns of training trajectories. Use of secondary test database provided a promising model pre-evaluation for the PHM08 data challenge that would otherwise be ambiguous due to the risk of one-time RUL estimate submission.

Considering the performance of the prognostic algorithms and their functions under complex system conditions, reconstructed databases can particularly provide enhanced information that will empower for any re-assessment and re-development of models. Additionally, the applications of prognostic frameworks can be validated in terms of whether they can perform in different scenarios or datasets representing different degradation patterns. High performance in a particular data set does not guarantee the same performance level in another data. As an example, the leading score for the PHM08 ‘test’ data could only rank $22^{\mathrm{nd}}$ on the ‘final test’ data leader board [2]. Since the run-to-failure trajectories of the training data are given and their true lifetime can be calculated from these patterns, reconstructed databases can be used to provide many similar but behaviorally different test scenarios for prognosis, and they can also serve a robust performance validation method for the evaluation of the accomplishments of prognostic frameworks prior to testing on the ‘final’ submission.

In the reconstructed databases*,* the total test trajectory unit number and their operational length are identical to those of the original “final test” data set. For each secondary test unit produced, a base case, $T_{b}$, is assigned from a randomly selected original training data.

(2) $$T_{b}={\mathrm{rand}(\arg}_{i=1,\ldots ,n}\mathrm{find}(L_{{\mathrm{tr}}_{i}}>L_{te}))$$

where *n* is the number of training trajectories, $L_{{\mathrm{tr}}_{i}}$ is a vector containing the lengths of original training units and $L_{\mathrm{te}}$ is the length of the ‘final test’ trajectory unit that is desired to be reconstructed. A randomly chosen location from the randomly selected training data, $T_{b}$ , is then taken off:

(3) $$S=\{\begin{array}{cc} rand\left(1:\left\lfloor \left(T_{\mathrm{tb}}-L_{te}\right)L_{1}\right\rfloor \right) & if\;L_{\mathrm{te}}<S_{1}\\ rand\left(1:\left\lfloor \left(T_{\mathrm{tb}}-L_{te}\right)L_{2}\right\rfloor \right) & if\;L_{\mathrm{te}}\geq S_{1}\;and\;L_{\mathrm{te}}<S_{2}\\ rand\left(1:\left(T_{\mathrm{tb}}-L_{te}\right)\right) & if\;L_{\mathrm{te}}\geq S_{2} \end{array}$$

where

*S* is the starting point
$S_{1}$ = 70 and $S_{2}$ = 100
$L_{1}$ = 1/2 and $L_{2}$ = 2/3

Selection process of start and end points for partial trajectories (“location” points) under secondary datasets should be reliable and performance-efficient: the original data were simulated by someone other than the researcher, and the test trajectories may include both larger and shorter samples of operations. Considering the prediction performance and prognostic metrics, the advantage of randomly selected data with a random operational index is that the impact of the participant is limited and the prognostic performance evaluation can be performed in multiple complex cases before the final submission. The randomly selected location can successfully model the potential high-risk cases, and the preliminary estimates of performance measurements for the “final test” subset can be improved by using pre-evaluated datasets. In the original “test” and “final test” subsets of PHM08 data, it was observed that the trajectories that were cut off early in time (short trajectories) were always in the initial and stable stages of the degradation. To be sure that a secondary data instance representing a short test trajectory can model an accurate baseline for primary research design, maximum ending points for random selection was set to limit the location accurately. In this way, the short secondary data trajectories could have a pre-established level of authenticity.

After the starting point (*S*) is calculated, the reconstructed ‘final test’ trajectory Ts is stored in the secondary dataset file.

(4) $$\mathrm{Ts}={T_{b}}_{\left(S:S+L_{te}\right)}$$

This means that the intensive interventions to the data are limited and all the secondary data test database values are originally sourced from the PHM08 dataset and C-MAPSS simulation.

Multiple secondary datasets are reconstructed and used in the pre-validation process for both filtering and prediction. As these datasets are generated from the original run-to-failure trajectories, their failure points as well as their exact true RULs are known. They are also made up of real scattered and noisy sensor values within multiple regimes, and moreover they form a very practical and suitable source for the developed model.

**Fig. 3:**
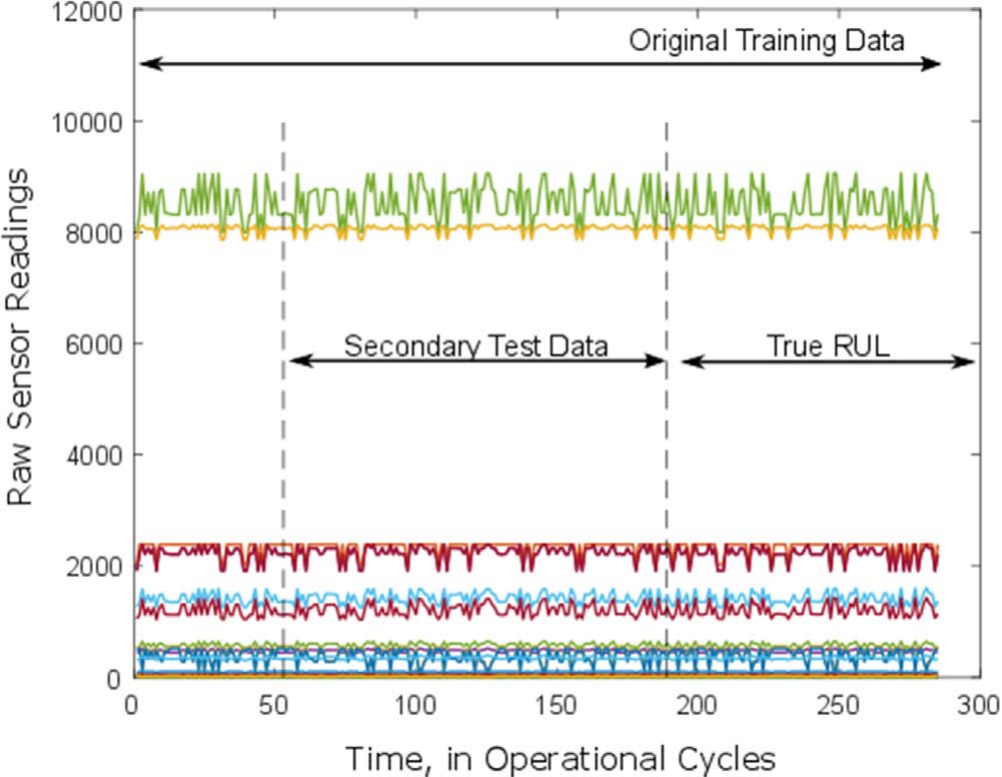
The principal start and end margins of secondary data.

In Fig. 3, a sample of the secondary data reconstruction is shown. The data location for test trajectory is defined by the start and end margins. As seen on the figure, the secondary test data are cut out from the training trajectories formed of run to failure training data in which the RUL is known as a ground truth. This implementation aims to illustrate the efficiency of the proposed model by using real degradation data from the C-MAPSS simulation. Based on the true remaining useful life and full operational behaviors in the plot, the prognostic-related behavior could be observed by using the complete degradation pattern for each test unit. The subsequent steps after the selected location is available from the base training data and has already been sourced from the same supplier, making it convenient to carry out further research.
