# Supplementary material for: Reconstructing secondary test database from PHM08 challenge data set
Source: Data Brief. 2018 Nov 20;21:2464–9. doi: 10.1016/j.dib.2018.11.085 (PMC6288980; doi:10.1016/j.dib.2018.11.085)
Supplement: Supplementary file 1 — Supplementary material [file mmc1.docx]

Conflict of Interest Form

The authors have declared that there is no conflict of interest.
